# Case Report: Abnormal pupils caused by the mitochondrial MT-TL1 gene m.3243A>G mutation

**DOI:** 10.3389/fped.2025.1573886

**Published:** 2025-08-21

**Authors:** Yujing Li, Yihong Ding, Enzhong Jin, Hong Yin

**Affiliations:** ^1^ Department of Ophthalmology, Peking University People's Hospital, Beijing, China; ^2^ Beijing Key Laboratory of Ocular Disease and Optometry Science, Peking University People's Hospital, Beijing, China

**Keywords:** m.3243A>G mutation, iris defect, infant, ocular screening, MT-TL1 gene

## Abstract

**Background:**

The m.3243A>G mutation in the MT-TL1 gene is the most common mtDNA mutation. The mutation can lead to a spectrum of conditions, including diabetes, hearing loss, heart and muscle involvement, encephalopathy and epilepsy, gastrointestinal problems, and vision impairment, often occurring concurrently—collectively referred to as MELAS (mitochondrial encephalopathy lactic acidosis and stroke-like episodes) syndrome. Currently, it has been reported that the ocular manifestations of m.3243A>G include posterior subcapsular cataract, ptosis, extraocular muscle paralysis, and retinitis pigmentosa, among which retinitis pigmentosa is the most common ocular manifestation.

**Methods:**

The ocular manifestations of a 10-month-old infant with mitochondrial MT-TL1 gene m.3243A>G mutation detected by genetic testing due to developmental delay were reported.

**Results:**

Ocular examination revealed Schiotz tonometry conversion values of 5.5/6 in the right eye (OD) and 5.5/4 in the left eye (OS) for intraocular pressure. Cycloplegic refraction measured +9.50 DS/−2.00 DC × 110° (OD) and +4.00 DS/−1.75 DC × 30° (OS). Anterior segment evaluation showed an irregular vertically oval pupil with absence of the temporal iris OD and a fusiform pupil OS, with no other anterior segment abnormalities detected in either eye. Fundus examination demonstrated clear optic disc boundaries bilaterally and a cup-to-disc (C/D) ratio of 0.3. Sodium fluorescein angiography revealed an intact retina without evidence of peripheral vascular leakage.

**Conclusions:**

Iris defect in a infant caused by m.3243A>G mutation was reported, which complements the ocular signs of this mutation and provides a new aspect for eye screening.

## 1 Introduction

MELAS (mitochondrial encephalopathy lactic acidosis and stroke-like episodes) syndrome is marked by involvement across multiple systems with neurological manifestations. It is a rare, inherited, neurodegenerative disorder caused by mitochondrial dysfunction that leads to energy production disturbances (1). Among patients with MELAS, 80% have a point mutation occurring at position 3,243 nucleotide (A3243G) in the mitochondrial MT-TL1 gene (2).

Ocular manifestations associated with the m.3243A>G mutation include posterior subcapsular cataracts, ptosis, ophthalmoplegia, and retinal pigmentary degeneration. Stroke-like episodes affecting the occipital cortex or other parts of the visual pathway can also lead to cortical blindness. Among these, retinal pigmentary degeneration is the most common ocular manifestation, ranging from 38% to 86% of cases (3). Current research has found that m.3243G is not randomly distributed, with high content in the neuroretina and RPE, but low content in the choroidal endothelium. The research also found that the presence of m.3243G leads to genetic dysregulation involved in oxidative stress management and the mTOR/PI3K/AKT signaling pathway (4).

However, in this case report, no signs of retinitis pigmentosa were observed. Instead, an isolated iris abnormality, a rare and noteworthy finding, was documented. There are few such reports, and this report can provide data supplement for the ocular manifestations of MELAS.

A 10-month female infant presented to the ophthalmology department at Peking University People's Hospital after her parents had noticed abnormal pupils for six months. The infant exhibited developmental delays and underwent genetic testing, which identified the mitochondrial mutation m.3243A>G. Neither parent was found to carry this mutation.

**Figure 1:**
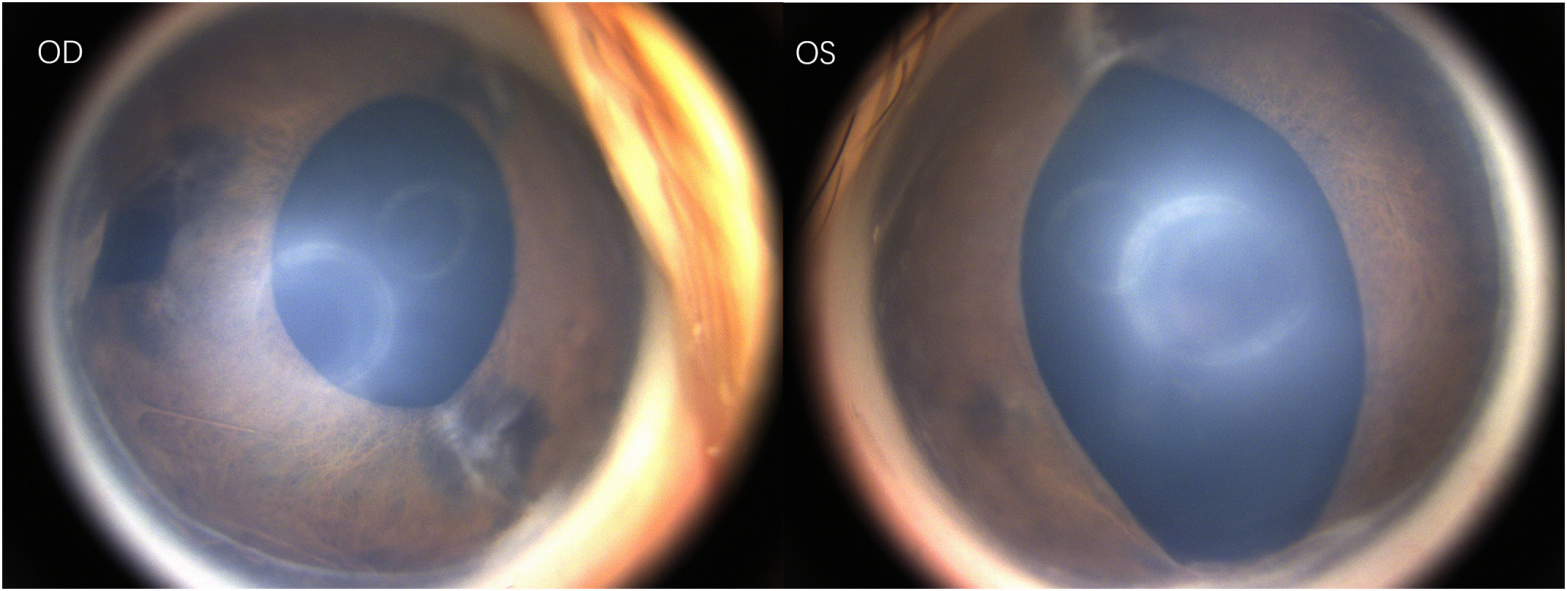
Photography of the anterior eye segment.

**Figure 2:**
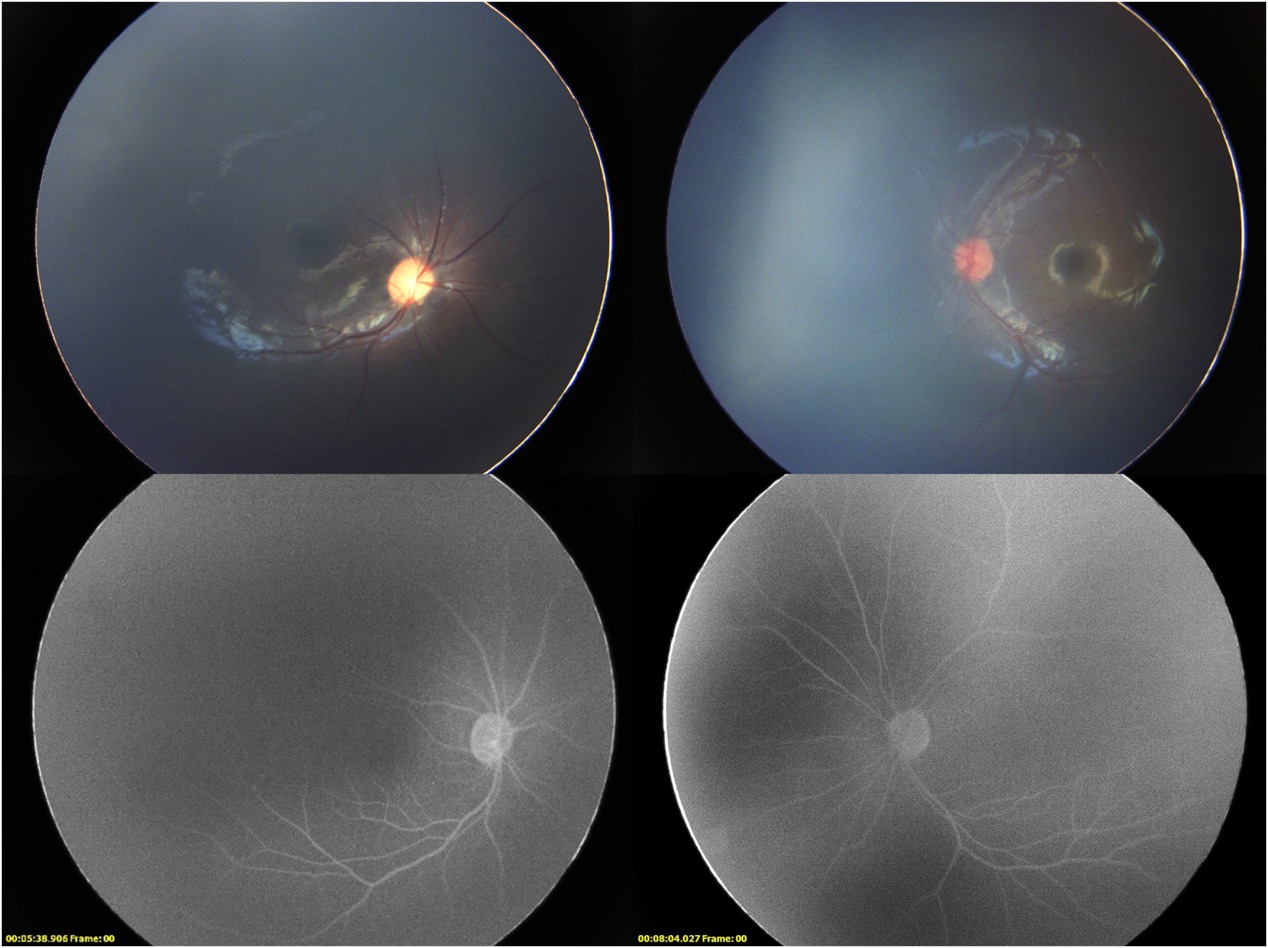
Binocular fundus imaging and late phase fluorescence angiography. The fluorescence angiography of the right eye was taken at 5 min 38 s, and the fluorescence angiography of the right eye was taken at 8 min 4 s.

Under general anesthesia, the ophthalmic examination revealed intraocular pressure measured by Schiotz tonometry: 5.5/6 in the right eye and 5.5/4 in the left eye. Refraction examination showed +9.50DS/−2.00DC × 110° in the right eye and +4.00DS/−1.75DC × 30° in the left eye. Both corneas were clear; the right pupil was vertically oval with temporal iris hypoplasia, and the left pupil was spindle-shaped. (Figure 1) Both lenses were clear, optic disc boundaries were sharp (cup-to-disc ratio = 0.3), and the retina was in position. Fluorescein angiography showed no peripheral vascular leakage (Figure 2).

## 2 Case description

The infant was born at term via natural delivery, weighing 3,300 grams, with an unremarkable pregnancy and no history of inhalation of oxygen or blood transfusions after birth. Parents noted “crossed eyes” at birth, which resolved within one month. By the time of the visit, the child had achieved several developmental milestones such as lifting her head at five months, rolling over at six months, and sitting at nine months. However, she had not yet begun to crawl and could only make sounds similar to “mama.” A neurological examination showed low muscle tone in her limbs. Her hands performed transverse palmar creases. The parents had not observed any ocular movement disorders at the time of the visit; although esotropia was previously suspected during outpatient evaluation, the child was uncooperative during the outpatient examination, making it difficult to assess the movement of the extraocular muscles. It is recommended that the parents closely monitor the child's ability to track objects. At this stage, it is advised that the child be fitted with glasses to prevent amblyopia resulting from high hyperopia.

## 3 Disscussion

Mitochondria are the only organelles, aside from the cell nucleus, that contain their own DNA (mtDNA). The m.3243A>G mutation in the MT-TL1 gene is the most common mtDNA point mutation. The prevalence of mitochondrial diseases is estimated to be approximately 23.3/100,000, while the prevalence of the m.3243A>G point mutation is 7.8/100,000. Epidemiological studies in China indicate that the m.3243A>G mutation accounts for 48.7% of mitochondrial diseases (5).

This mutation can lead to a range of conditions, including diabetes, hearing loss, cardiac and muscular dysfunction, encephalopathy and epilepsy, gastrointestinal issues, and visual disturbances. These symptoms often manifest collectively as MELAS syndrome. Diabetes is the most common endocrine manifestation, with a prevalence of 40%–50% among all carriers of the m.3243A>G mutation. Patients with this mutation tend to be relatively younger and exhibit a combination of insulin resistance and deficiency. Gastrointestinal complications affect over 75% of patients, with early-onset disease frequently manifesting as growth retardation, malnutrition, and weight loss. Symptoms include nausea and vomiting, constipation, bloating, and abdominal distension. Maternal hereditary diabetes and deafness are the most common phenotypes associated with the m.3243A>G mutation, occurring in approximately 30% of cases (6, 7). The molecular mechanism by which the m.3243 A>G mutation affects insulin secretion may involve the attenuation of cytosolic ADP/ATP levels, leading to a resetting of the glucose sensor in pancreatic β-cells. Age-dependent deterioration of pancreatic function in carriers of the m.3243A>G mutation is attributed to changes in ATP and reactive oxygen species levels (8).

Single-cell sequencing of MELAS retinal tissue demonstrates that cellular responses to m.3243A>G heteroplasmy involve mTOR pathway activation. Multimodal analysis of retinal pigment epithelium (RPE) reveals that high mutation burden correlates with RPE dysfunction, representing an early stage in MELAS-associated retinopathy in MELAS (4).

It was demonstrated that the m.3243A>G mutation leads to the inhibition of mitochondrial autophagy, accompanied by changes in lysosomal homeostasis, upregulation of glycolysis, increased reactive oxygen species (ROS) generation, and redox imbalance (9, 10). Lysosomal damage induced by mitochondrial dysfunction or ROS can be categorized into two scenarios: lysosomal enlargement and increased lysosomal numbers, because of chronic mitochondrial defects, and increased lysosomal membrane permeability and cell death due to acute ROS production (9). A deficiency of ubiquinone oxidoreductase (Complex I) is a common feature in biochemical and pathological studies related to m.3243A>G-associated MELAS. When Complex I is impaired, NAD+ levels decrease, NADH accumulates in cells, and ATP production decreases. NADH overload is associated with ROS generation, blockage of the tricarboxylic acid cycle, damage to the synthesis of aspartate, and disruption of transcriptional regulation (11).

Chih-Yao Chung et al. identified aberrant activation of the PI3K-Akt-mTORC1 pathway in m.3243A>G mutants, increasing glucose uptake and dependence, and enhancing lipid synthesis, leading to redox imbalance, which is particularly characteristic of the m.3243A>G mutation (12). Inhibitors targeting PI3K, Akt, or mTORC1 reduced the abnormal ROS load in the mutants and partially restored mitochondrial bioenergetic function, decreasing glucose dependence. These findings suggest the PI3K-Akt-mTORC1 axis as a potential therapeutic target for this type of mitochondrial disease.

Case reports of m.3243A>G mutation are mostly associated with MELAS syndrome, while reports of ocular signs and symptoms are relatively rare, and mainly focus on cases of retinitis pigmentosa in adults and ptosis and strabismus in adolescents. Retinal pigmentary degeneration associated with this mutation is more frequently observed in female patients. A meta-analysis in 2021 found that patients with m.3243A>G variant-related pigmentary retinopathy maintain good vision before the age of 50, after which their vision significantly declines. Patients without hearing loss or diabetes are less likely to develop retinal degeneration, which typically manifests about 10 years after the diagnosis of hearing loss and diabetes. Extensive deletions in mtDNA are associated with widespread retinal dysfunction and diffuse pigmentary retinopathy, while point mutations, such as m.3243A>G, are more commonly associated with more localized macular pigmentary and atrophic changes. In these cases, fundus abnormalities are most prominent around the fovea and adjacent areas, with yellow-white spots appearing most prominently in the nasal and temporal area of the optic disc on fundus autofluorescence. In the early to mid-stage of the disease, the foveal structure and function remain preserved, with minimal impact on vision; however, foveal involvement in later stages results in significant visual deterioration. Early-stage manifestations of the m.3243A>G mutation include thickening of the outer segment tips and the ellipsoid zone, while late-stage features including xanthomas, indicates mitochondrial-induced photoreceptor damage (3).

For patients with ptosis and ophthalmoplegia, muscle histology shows typical “ragged red fibers,” which are indicative of secondary mitochondrial proliferation. During ptosis correction surgery, it may be beneficial to perform a biopsy of the orbicularis oculi muscle, as this can provide diagnostic insights while avoiding biopsies from other sites, thereby reducing costs and morbidity (13). Kearns-Sayre syndrome is a specific form of chronic progressive ophthalmoplegia accompanied by other lesions not related to the muscular system, presenting severe multisystem phenotypes before the age of 20. Its features include ptosis, ophthalmoplegia, retinal degeneration, cardiac conduction block, cerebellar ataxia, and elevated cerebrospinal fluid protein (3).

In 1993, Rummelt et al. described the clinical, histopathological, and ultrastructural findings of two eyes obtained from an autopsy of a 21-year-old female patient with MELAS syndrome (14). The patient presented with bilateral ptosis, chronic ophthalmoplegia, diffuse choroidal atrophy, atypical pigmentary retinopathy with macular involvement, and patchy atrophy of the iris stroma. Molecular genetic analysis detected a point mutation in the tRNA Leu (UUR) at nucleotide 3,243 of mitochondrial DNA. Histological and ultrastructural examinations revealed atrophy of the iris stroma, with numerous structurally abnormal mitochondria, typically enlarged, present in the dilator muscle of the iris, sphincter, cornea, lens epithelium, and ciliary epithelium. The pathological finding was considered related to secondary mitochondrial proliferation based on current researches.

In 2010, a retrospective study was conducted on ocular-related symptoms in 59 patients diagnosed with mitochondrial diseases during childhood and adolescence, with 12 cases related to this m.3243A>G mutation (15). The youngest patient was 4 years old, presenting only refractive errors. As patients aged, ocular manifestations gradually increased. In patients under 12, the main ocular symptoms included ptosis, strabismus, nystagmus, uncorrected visual acuity, and refractive errors. Patients aged 15 and 18 exhibited optic atrophy, while another 15-year-old patient and several patients over 18 were observed retinal pigmentary degeneration. No cases with an earlier onset of the m.3243A>G mutation have been reported.

Currently, among the reports on ocular abnormalities caused by the m.3243A>G mutation, only Reports of ocular abnormalities associated with the m.3243A>G mutation rarely include iris atrophy, with the exception of Rummelt's 1993 report involving an adult patient (14). In the present case, the patient is younger and has not shown bilateral ptosis during the ophthalmic examination. Iris atrophy could be explained by abnormal muscle with mitochondrial proliferation. Aside from iris atrophy, no abnormalities were observed in the cornea, lens, or retina, and there is no evidence of optic nerve atrophy. The child is developmentally delayed. The head MRI examination can be conducted to assess whether the cortex is involved. In MELAS syndrome, oxidation-reduction reactions are abnormal due to mitochondrial abnormalities, leading to abnormal accumulation of lactic acid, abnormal microvascular pressure, abnormal ion concentrations in cells, and potentially causing metabolic acidosis-like reactions or seizures. These factors may contribute to structural abnormalities in the cerebral cortex and developmental delays (16).

As mentioned before, antioxidant therapy can theoretically prevent oxidative damage caused by ROS (11). Some studies have attempted gene therapy for Leber's Hereditary Optic Neuropathy (LHON), another disease related to m3243A>G mutation, through intravitreal injection of adenoviruses, which resulted in improved vision in patients (17, 18). This has offered new hope for the treatment of mutations related to m3243A>G.

This case report has some limitations. Further ophthalmic examinations such as optical coherence tomography (OCT) examination, visual field examination and vision examination could not be obtained because the child was too young to cooperate. In addition, this patient should be followed for future ocular manifestations to see if other ocular manifestations develop as she ages.

Therefore, for patients with the m.3243A>G mutation detected by postnatal genetic screening or diagnosed with MELAS syndrome, regular eye screening is essential. Significant vision loss or nystagmus may not be observed in patients with isolated iris defect, which may prevent them from ocular examination. For example, the child in this case currently presents with iris defect and high hypermetropia. Without timely correction of hypermetropia, visual development may be impaired, even if there are no related complications in the fundus. The only prior case report of iris defects of this mutation in adults was published by Rummelt in 1997, but it is possible that some cases with the ocular signs of this mutation have not been documented because their vision wasn't influenced and they didn't take an eye examination.

## 4 Conclusion

This case report presents a 10-month-old infant with the mitochondrial MT-TL1 gene m.3243A>G mutation manifesting distinct iris abnormalities and high hyperopia, which expands the known ocular manifestations of this mutation, highlights the need for early ocular screening in such cases to enable timely intervention and monitoring.

## Data Availability

The original contributions presented in the study are included in the article/Supplementary Material, further inquiries can be directed to the corresponding authors.
